# Senescent Mesenchymal Stromal Cells Drive Stem Cell Aging Through Dysregulated Chemokine Synthesis

**DOI:** 10.1111/acel.70780

**Published:** 2026-10-28

**Authors:** Shaianne N. Stein, Lorena R. Braid

**Affiliations:** ^1^ Department of Molecular Biology and Biochemistry, Centre for Cell Biology, Development and Disease Simon Fraser University Burnaby Canada

**Keywords:** aging, chemokines, inflammaging, mesenchymal stromal cells, rejuvenation, senescence, stem cells

## Abstract

Aging is accompanied by a progressive decline in tissue regenerative capacity, driven by intrinsic stem cell dysfunction and age‐associated deterioration of the surrounding stem cell niche. Mesenchymal stromal cells (MSCs) are ubiquitous components of stem cell niches, where they integrate structural, metabolic, inflammatory, and paracrine cues to regulate stem cell quiescence, activation, and differentiation. With age, MSCs undergo functional decline and senescence, altering their secretory and signaling profiles. Across tissues, MSC senescence emerges as an intermediate amplifier of aging: molecular, metabolic, epigenetic, and inflammatory stressors promote the senescent state, which converts these upstream insults into chemokine imbalance, chronic inflammation, and progressive loss of stem cell support. Here, we synthesize classical and emerging literature to examine how age‐associated changes in MSC chemokine synthesis contribute to stem cell aging, and we propose a synthesis‐based model in which MSCs translate age‐associated stressors into altered chemokine repertoires that disrupt stem cell‐supportive signaling. We also discuss therapeutic strategies, including chemokine‐directed therapies, senolytics, and MSC rejuvenation, that may restore regenerative capacity by normalizing MSC‐derived signals. Together, the evidence identifies dysregulated chemokine synthesis as a conserved mechanism linking tissue‐resident MSC aging to declining regenerative capacity.

## Graphical abstract

Senescent MSCs do more than fuel inflammaging: they disrupt the chemokine signals that maintain stem cell function. We propose that restoring this signaling balance through senolytics, senomorphics, or MSC rejuvenation may preserve regenerative capacity during aging.

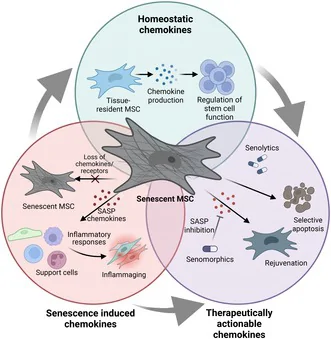

## 1 Introduction

Regeneration is a foundational determinant of biological longevity. Over a lifetime, higher organisms sustain continuous biochemical, molecular, and cellular damage. In the absence of efficient repair and replacement mechanisms, damaged and exhausted cells accumulate, leading to progressive tissue dysfunction. Thus, human aging is a consequence of compounded cellular damage that outpaces the body's capacity for renewal. This reparative capacity varies across tissues; some depend on continuous stem cell‐mediated replacement, whereas others, including the brain and skeletal muscle, rely mainly on long‐lived post‐mitotic cells.

Stem cells provide replacement cells for tissue maintenance and repair. Their behavior is regulated by physical, mechanical, biochemical, and multicellular cues within specialized niches (Atcha et al. 2023; Hicks and Pyle 2023; C. Li et al. 2024; Luo et al. 2022; Shafiq et al. 2021). Aging erodes niche components, impairing stem cell regulation and tissue organization (Farahzadi et al. 2023; Fuchs and Blau 2020). Dysregulated chemokine secretion contributes directly to stem cell exhaustion across tissues (Chaudhary et al. 2025; Fu et al. 2025; Kang et al. 2026; Mi et al. 2022), making the aging niche a therapeutic target for improving regeneration.

The niche microenvironment protects stem cell reservoirs and provides instructive cues to stem cells and their progeny (Farahzadi et al. 2023; C. Li et al. 2024). Although niche architecture and composition vary among tissues, core mechanisms governing quiescence, self‐renewal, proliferation, and differentiation are conserved (Fuchs and Blau 2020; Mannino et al. 2022; Urbán and Cheung 2021; van Velthoven and Rando 2019). One common feature is the presence of mesenchymal stromal cells (MSCs), which serve as essential organizing and support cells within many stem cell niches and across perivascular and stromal compartments (Gan et al. 2022; B. Li et al. 2023; Pilato et al. 2018; Sagaradze et al. 2020; B. Wang et al. 2020).

The broad distribution and environmental responsiveness of MSCs may translate age‐associated stress into altered stem cell behavior across tissues. Here, we propose a synthesis‐based model of stem cell aging in which signals produced by supportive niche cells link changes in the aged tissue environment to neighboring stem cell function. This model asks whether age‐dependent MSC signaling, particularly altered chemokine synthesis, helps explain regenerative decline across stem cell systems. We focus on chemokines because they are major SASP components and regulate immune recruitment and injury responses while recognizing that loss of trophic support, extracellular matrix (ECM) remodeling, and other mechanisms also contribute.

### 1.1 Mesenchymal Stem/Stromal Cells

MSCs were first identified in bone marrow (BM) by their fibroblast‐like morphology and clonogenic potential (Friedenstein et al. 1968) and later shown to undergo osteogenic, chondrogenic, and adipogenic differentiation (Friedenstein et al. 1974). MSCs have since been described in nearly all adult tissues and organs (da Silva Meirelles et al. 2006) and in perinatal tissues, demonstrating broad distribution (Allouh et al. 2025; Araújo et al. 2017; Fukuchi et al. 2004; H.‐S. Wang et al. 2004). Despite extensive ex vivo characterization, MSC identity, heterogeneity, and in vivo functions remain incompletely defined (Girousse et al. 2021; Kurosawa et al. 2024; Pittenger et al. 2019; Soliman et al. 2021).

The term “MSC” distinguishes culture‐expanded cells from putative counterparts in native tissues. Once expanded ex vivo, human MSCs are defined by plastic adherence, CD73, CD90, and CD105 expression, absence of CD45, CD34, CD14 or CD11b, CD79α or CD19, and HLA‐DR, and osteogenic, adipogenic, and chondrogenic differentiation in vitro (Dominici et al. 2006). In vivo, no universal marker set exists. Putative MSCs are identified by tissue‐specific localization, surface markers, lineage tracing, and functional assays. CD146‐positive adventitial reticular cells in human BM can self‐renew and recreate a hematopoietic microenvironment after transplantation, whereas CD146‐, NG2‐, and PDGFRβ‐expressing perivascular cells from multiple organs acquire MSC‐like properties in culture (Crisan et al. 2008; Sacchetti et al. 2007). However, lineage tracing shows that pericytes in several organs do not broadly generate tissue‐specific mesenchymal lineages during homeostasis, limiting their general classification as MSCs (Guimarães‐Camboa et al. 2017). Throughout this review, “MSC” therefore denotes tissue‐resident mesenchymal stromal populations with reported MSC‐like phenotypes or functions, without implying a common developmental origin, marker profile, or multipotency.

Many in vivo populations described as MSCs reside in basement membrane‐associated and perivascular compartments, where they regulate architecture, signaling, and inflammation. Their position at the blood vessel–tissue interface enables integration of systemic and local cues that regulate tissue homeostasis and repair (Benabid and Peduto 2020; Wu et al. 2020). After injury or infection, MSCs can mobilize to damaged sites and act as “first responders” that regulate immune activation and repair (Caplan 2017a, 2017b; Caplan and Dennis 2006; Hori et al. 2024; Sajjad et al. 2024). In response to microenvironmental cues, MSCs secrete context‐specific growth factors, cytokines, and chemokines that initiate pro‐ or anti‐inflammatory repair (Burja et al. 2020; English 2013; Leuning et al. 2018; Sarsenova et al. 2022; Song et al. 2020). In young tissues, MSC‐derived chemokines regulate stem cell localization and function. With age, MSC senescence and functional decline can convert these adaptive signals into persistent dysregulation. We therefore review how aberrant chemokine synthesis by tissue‐resident MSCs may reduce regenerative capacity with age.

### 1.2 Aging and Senescence

Age strongly predicts chronic and degenerative diseases, including diabetes, cardiovascular disease, osteoarthritis, and cancer (Chia et al. 2018; Coppé et al. 2008; Ghosh et al. 2020; Schosserer et al. 2017). Chronological age is time since birth; biological age reflects accumulated physiological damage and better predicts longevity and disease susceptibility (Ukraintseva et al. 2021; J. W. Wu et al. 2021). In vitro, serial passage produces replicative aging or senescence (Wiese et al. 2019).

The hallmarks of biological aging are interconnected rather than parallel processes. Damage‐generating changes, including genomic instability, telomere attrition, loss of proteostasis, and epigenetic alterations, interact with metabolic and mitochondrial dysfunction to promote senescence. Senescence then alters intercellular communication and reinforces chronic inflammation, contributing to stem cell exhaustion (López‐Otín et al. 2013, 2023). In addition, aging is accompanied by chronic, low‐grade inflammation, termed inflammaging, which both drives and is reinforced by progressive cellular dysfunction (Ferrucci and Fabbri 2018; Saavedra et al. 2023).

Cellular senescence is stable mitotic arrest in metabolically active cells that no longer perform normal physiological functions. It limits the proliferation of damaged cells and helps to prevent malignant transformation. Senescent cells secrete growth factors, proteases, cytokines, and chemokines collectively termed the senescence‐associated secretory phenotype (SASP) (Basisty et al. 2020; Coppé et al. 2010; Ohtani 2022).

During embryonic development and wound healing, acute senescence limits proliferation and fibrosis while promoting tissue remodeling (Dodig et al. 2019; Huang et al. 2022; Langhi Prata et al. 2018; Meng et al. 2020; Schafer et al. 2017; Storer et al. 2013). SASP factors then recruit immune cells that clear senescent cells and enable regeneration (Birch and Gil 2020; Jang et al. 2026; Kale et al. 2020). Chronic senescence instead arises from accumulated damage and SASP profiles that evade immune surveillance, allowing senescent cells to persist (Jang et al. 2026; K.‐A. Lee et al. 2022). Acute and chronic senescence are reviewed elsewhere (Childs et al. 2015; Heath and Grant 2020; van Deursen 2014).

Senescent cell accumulation is linked to age‐associated functional decline and disease (Allen et al. 2022; Yousefzadeh et al. 2020). Systemic pro‐inflammatory SASP components are hallmarks of aging and contribute to immune dysregulation and chronic disease (Chaudhary et al. 2025; Furman et al. 2019; Lopes‐Paciencia et al. 2019). Transplanting senescent cells into young mice induces systemic decline (Xu et al. 2018), directly supporting a causal role in aging (Allen et al. 2022; Wissler Gerdes et al. 2021; Yeo et al. 2021).

Senescent MSCs thus act primarily as niche‐level amplifiers of aging. MSC senescence converts upstream molecular, metabolic, and inflammatory stress into lost homeostatic support and a chemokine‐rich SASP (Figure 1). These signals can propagate paracrine senescence, recruit or reprogram immune populations, and reinforce chronic inflammation (Chou et al. 2022; Gnani et al. 2019). Senescent MSCs may secondarily drive stem cell exhaustion by disrupting quiescence, self‐renewal, differentiation, and niche organization, as shown most directly in the hematopoietic compartment (Gnani et al. 2019; O'Hagan‐Wong et al. 2016).

**FIGURE 1:**
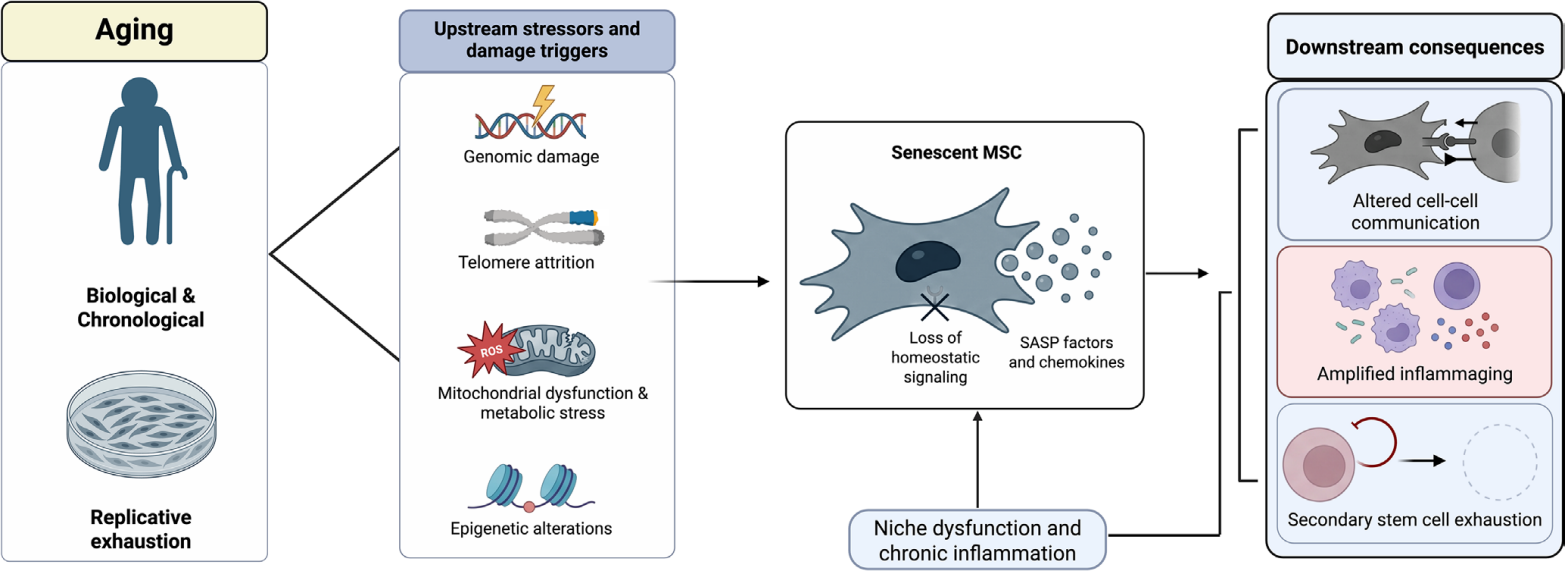
Senescent MSCs drive a feed‐forward loop. Whether senescence occurs biologically in vivo or through replication in vitro, MSCs translate molecular, metabolic, and epigenetic stress into disrupted homeostasis and niche regulation. These signals promote paracrine senescence, alter stem and supportive niche cell behavior, and reinforce chronic, low‐grade inflammation. Figure created in BioRender.

### 1.3 Chemokine Synthesis as a Mediator of Stem Cell Aging

Chemokines are essential signaling factors that mediate communication between stem cells and niche populations. These 8–12 kDa ligands signal mainly through G protein‐coupled receptors (Chaudhary et al. 2025). Receptor promiscuity fine‐tunes signal strength and spatial localization. Chemokine–receptor gradients control stem cell positioning, quiescence, activation, and mobilization within niches (Lane et al. 2014). During chronic inflammation and aging, dysregulated chemokine signaling disrupts niche integrity and stem cell quiescence, activation, and mobilization (Wagers 2012). This dysregulation can reflect excessive ligand production or age‐dependent receptor loss on responding cells (Coppé et al. 2010; Wagers 2012). Chemokines are major SASP components, and sustained elevation is linked to biological aging (Chaudhary et al. 2025).

MSCs are a relevant source of age‐dependent chemokine signals because they are broadly distributed within stem cell niches and respond to local and systemic cues. Aging progressively alters the chemokine repertoire synthesized by stem and support cells, including MSCs, changing the signaling environment of neighboring stem cells. Senescent MSCs convert upstream molecular, metabolic, epigenetic, and inflammatory stress into altered chemokine output, reinforcing niche dysfunction through a feed‐forward cycle. Thus, MSC senescence is both a consequence of molecular aging and an amplifier of downstream stem cell dysfunction.

## 2 Senescence Reprograms MSC Chemokine Synthesis

MSC senescence during cell manufacturing and therapy has been reviewed elsewhere (J. Liu et al. 2020; Lyamina et al. 2023; W. Sun et al. 2023; Y. Zhang et al. 2021). Here, we focus on tissue‐resident MSCs and their distinct implications for stem cell aging. Aged MSCs display canonical senescence markers, including increased p16 and p21, senescence‐associated β‐galactosidase (SA‐β‐gal) activity, and senescence‐associated heterochromatin foci (Figure 2) (Block et al. 2017; Gnani et al. 2019; M. Li, Guo, et al. 2024; Y. Liu and Chen 2020; Weng et al. 2022). Their SASP contains pro‐inflammatory chemokines, including IL‐8/CXCL8, CCL20, CXCL5, CXCL13, CCL26, and GRO family members. Compared with non‐senescent MSCs, SASP‐positive MSCs exhibit reduced immunomodulatory activity and create a pro‐inflammatory microenvironment (Chou et al. 2022; Gnani et al. 2019; Turinetto et al. 2016; X. Zhou et al. 2020). This altered secretome can propagate senescence to neighboring MSCs, induce inflammatory gene expression, and impair clonogenic function in hematopoietic stem and progenitor cells (Chou et al. 2022; Gnani et al. 2019).

**FIGURE 2:**
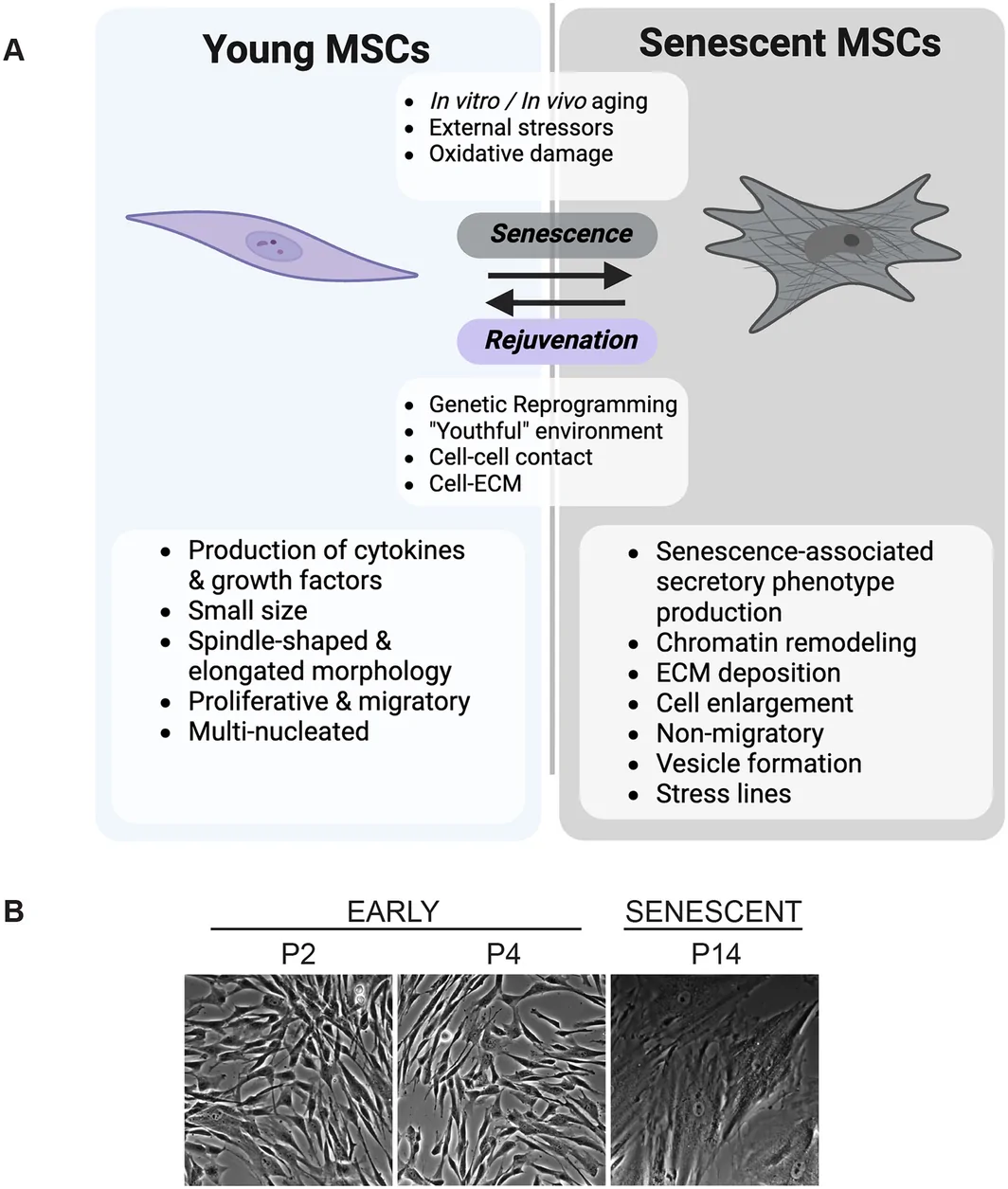
MSCs exhibit measurable age‐related changes. (A) Young and senescent MSCs exhibit morphological, secretome, and transcriptome differences. Figure created in BioRender. (B) Cultured MSCs illustrate the visible shift from young (P2) spindle‐like cells to enlarged, angular pre‐senescent cells (P14) (adapted with permission from Wiese et al. 2019).

Senescent MSCs also undergo characteristic morphological and functional changes (Figure 2). Increased size and granularity, prominent actin stress fibers, nuclear enlargement, and condensed nucleolin produce the characteristic “fried‐egg” morphology (Christodoulou et al. 2013; Massaro et al. 2023; Wagner et al. 2010; Weng et al. 2022; Wiese et al. 2019). By skewing innate and adaptive immune signaling, senescent MSCs reinforce chronic, low‐grade inflammation and contribute to inflammaging (Ferrucci and Fabbri 2018; Franceschi et al. 2018; Franceschi and Campisi 2014; B. C. Lee and Yu 2020). Age‐dependent downregulation of chemokine and cytokine receptors further reduces their responsiveness to activating paracrine signals, compounding this altered signaling phenotype (Figure 3, Table 1).

**FIGURE 3:**
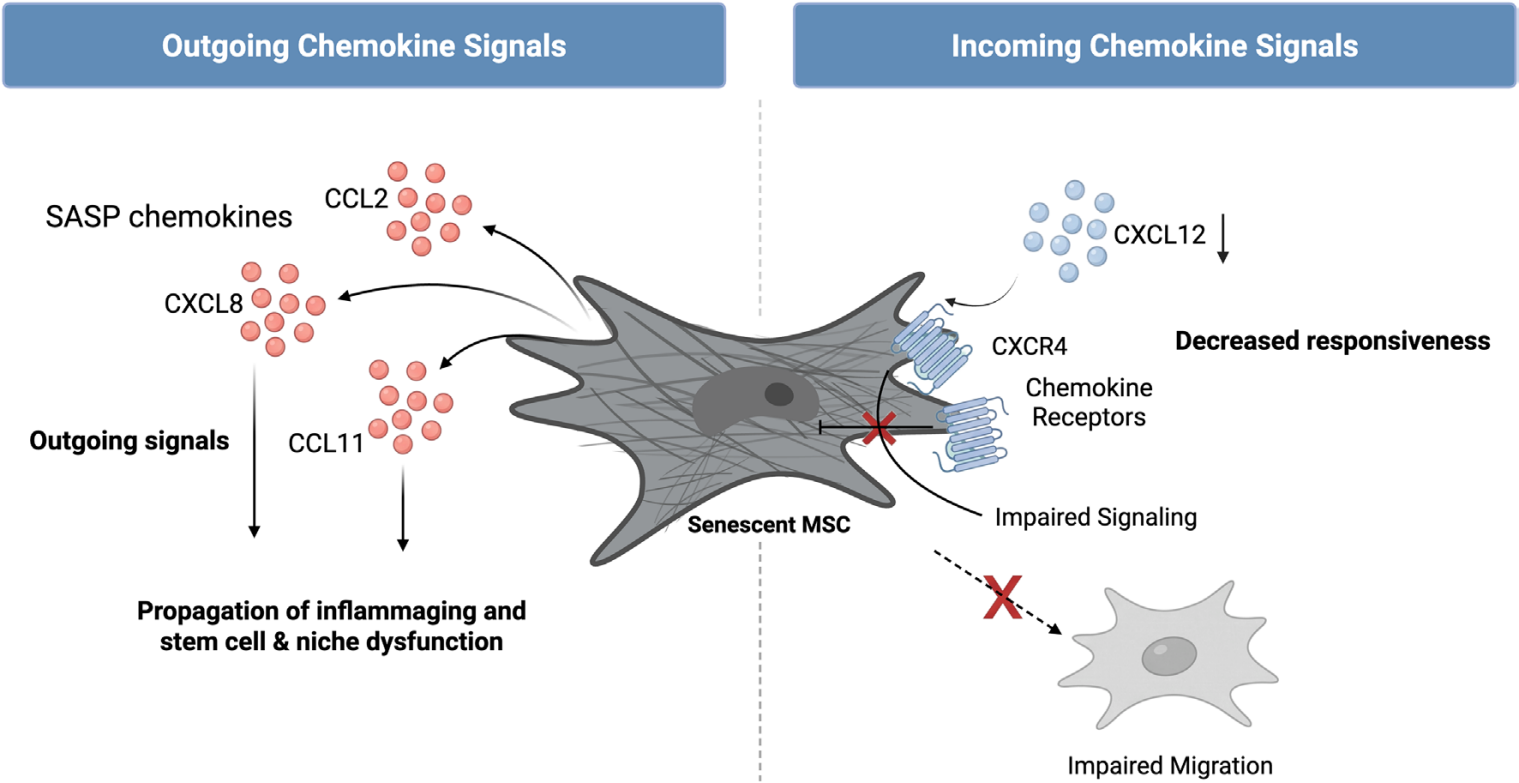
MSC senescence alters chemokine signaling. Senescent MSCs alter chemokine synthesis and secretion, creating a pro‐inflammatory milieu. They also become less responsive to incoming chemokine signals, disrupting homeostasis and promoting niche dysfunction. Figure created in BioRender.

**TABLE 1:** Mechanistically conserved MSC chemokine axes and relevant niches.

Chemokine axis: ligand—receptor	Chemokine function Supporting references	Defining characteristic(s)	Relevant stem cell niche(s) Supporting references
***CXCL12‐CXCR4***	MSC mobilization and homing, adhesion, in response to tissue injury, inflammation; decreased in aging (Cuesta‐Gomez et al. 2021; Guang et al. 2013; Hocking 2015; Kyriakou et al. 2008; Sanghani‐Kerai et al. 2017; Shao et al. 2011)	Homeostatic Senescence induced Therapeutically actionable	Bone marrow (Guang et al. 2013; Singh et al. 2020) Cranial suture (B. Li et al. 2023, 2025) Neural compartment (Zhu et al. 2019)
***CCL11*** * **‐CCR3**	MSC homing to cancer cells expressing receptor (CCL26/Eotaxin‐3), Protection from senescence with CCR3 antagonist (CCL11/Eotaxin‐1) (Ishida et al. 2018) (**Zayed et al.** 2020)	Homeostatic Senescence induced Therapeutically actionable	Dental pulp (**Zayed et al.** 2020) Lung tissue (Chanda et al. 2021) Skeletal muscle (Ambrosi et al. 2021)
***CXCL8/IL‐8***—**CXCR1, 2**	Potent chemoattractant signal received by MSCs; Main SASP increased during aging and during chronic inflammation. CXCL8 secreted by MSC supports survival of cancer cells (J. Wang et al. 2015) (Cheng et al. 2019)	Senescence induced Therapeutically actionable	Hair follicle (**Pappalardo et al.** 2025)
***CCL2*** * ***‐CCR2***	MSC enhancement of chemotaxis of activated T‐cells during inflammation, immune response (Y. Zhang et al. 2021)	Homeostatic Senescence induced Therapeutically actionable	Hair follicle (W. Wu et al. 2023) Skeletal muscle (Blanc et al. 2020)

*Note:* Studies are classified by chemokine function (homeostatic, senescence‐induced, and/or therapeutically actionable) and organized by stem cell niche. Studies using human systems are shown in bold.

* MSCs express the chemokine but not the associated receptor.

Together, these changes suggest that MSC senescence alters both sides of chemokine‐mediated communication: the repertoire of chemokines synthesized and secreted by MSCs and their capacity to sense and respond to extracellular signals. Within the synthesis‐based model proposed here, this shift is particularly important because MSCs are not passive recipients of age‐associated changes in the tissue environment. Rather, their transition to a senescent state can transform the signals they produce, communicating MSC cellular aging to surrounding stem and progenitor cells. Chemokines are normal components of MSC‐mediated tissue homeostasis and repair, and transient production of inflammatory chemokines can facilitate immune recruitment and regenerative responses. Importantly, the consequence of MSC senescence is not simply increased production of SASP chemokines, but also a loss of appropriate regulation of the identity, abundance, duration, and context of chemokine signals (Figure 3). Persistent production of SASP‐associated chemokines could thereby instigate and amplify an imbalanced chemokine signaling environment that progressively compromises stem cell function.

### 2.1 Dysregulated MSC Chemokine Synthesis Leads to Loss of Stem Cell Regulation

Aging is associated with loss of self‐renewal and depletion of the stem cell pool, accompanied by diminished differentiation capacity, altered metabolic and epigenetic states, and increased susceptibility to senescence‐inducing stressors (Khong et al. 2019; L. Liu et al. 2022; Weng et al. 2022; X. Zhang et al. 2026). Accumulating evidence indicates that niche‐resident support cells, including MSCs, are among the most vulnerable targets of aging (Brunet et al. 2023; Dong et al. 2026; B. Li et al. 2023; Mi et al. 2022; J. Oh et al. 2014; Rando et al. 2025).

Consequently, stem cell aging cannot be solely considered a cell‐intrinsic process but must also reflect age‐associated changes in the signals produced by the surrounding cellular environment. In our synthesis‐based model, MSCs are an important mechanistic link between these processes. Depletion of functional MSCs with age can reduce the availability of homeostatic signals required for stem cell maintenance, while accumulation of senescent MSCs introduces a second, complementary perturbation through aberrant synthesis of SASP‐associated chemokines. The resulting imbalance reflects both a loss of supportive signals and a gain of persistent or inappropriate signals, thereby altering the chemokine environment experienced by neighboring stem and progenitor cells.

This model does not imply that MSC senescence is the sole or initiating cause of stem cell exhaustion. Stem cells also accumulate substantial cell‐intrinsic damage with age. Rather, senescent MSCs can accelerate or stabilize exhaustion by superimposing persistent extrinsic chemokine signals and loss of homeostatic niche support upon intrinsically aged stem cells. This distinction matters because chemokine dysregulation need not involve a uniform increase in chemokine abundance. Aging can simultaneously deplete chemokines required for stem cell retention and increase inflammatory and chemotactic signals that drive inappropriate activation or immune‐cell recruitment. These effects are not limited to direct MSC–stem cell communication. Chemokines also recruit and position immune and other niche‐resident populations; therefore, altered MSC secretion can propagate through the broader cellular microenvironment and reinforce chronic inflammation.

The following sections examine evidence for this model across tissues and identify chemokine signaling programs that recur across otherwise distinct regenerative systems.

### 2.2 Homeostatic, Senescence‐Associated, and Therapeutically Actionable MSC Chemokine Programs

Across tissues, age‐associated changes in MSC chemokine synthesis can be organized into three intersecting functional programs: homeostatic chemokine programs that support stem cell maintenance and tissue regeneration; senescence‐associated chemokine programs that emerge as MSCs age or acquire senescent features; and therapeutically actionable chemokine pathways that can potentially be manipulated to restore regenerative function (Table 1). These categories are not mutually exclusive. The first two describe functional states of MSC signaling, whereas the third describes the potential to modify either state therapeutically. A single chemokine may therefore perform a homeostatic function when appropriately regulated, acquire detrimental effects when its abundance or persistence changes with age, and become therapeutically actionable when modulation of the ligand, receptor, or cellular source improves regenerative function. Because the evidence varies across tissues and models, Table 2 classifies each example as direct causal evidence, functional co‐culture or transplantation evidence, single‐cell or spatial association, or indirect inference.

**TABLE 2:** Expression of senescence‐associated chemokines across MSC stem cell niches.

Chemokines	Expression	Evidence	Niche	MSC type	Reference(s)
**Bone marrow and skeletal system**					
*CCL2, CCL3, CXCL12, CXCL16*	↑	**Indirect:** Xenograft transplantation; **Transcriptome SASP profiling (H)**	Bone marrow	Bone marrow MSC (senescent tissue‐resident, chronologically aged)	(**Khong et al.** 2019)
*CXCL10*	↓	**Indirect:** Transcriptome SASP profiling	Bone marrow	Bone marrow MSC (senescent tissue‐resident, chronologically aged)	(**Gnani et al.** 2019)
IL‐8/CXCL8, CCL4, CXCL2/GRO‐β, CCL2/MCP‐1	↑	**Indirect:** Transcriptome SASP profiling and protein quantification	Bone marrow	Bone marrow MSC (senescent tissue‐resident, chronologically aged)	(**Gnani et al.** 2019)
CXCL12‐CXCR4	↓	**Direct:** Functional co‐culture with young HSC	Bone marrow	Bone marrow MSC (senescent tissue‐resident, chronologically aged)	(Singh et al. 2020)
*Cxcl12/*SDF‐1	↓	**Indirect:** Transcriptome SASP profiling and protein levels Single‐cell RNAseq	Bone marrow Cranial suture	Bone marrow MSC (senescent tissue‐resident, chronologically aged) Cranial suture MSC (senescent tissue‐resident, chronologically aged)	(Guang et al. 2013) (B. Li et al. 2025)
**Skeletal muscle**					
*Ccl2, Ccl7*	↑	**Indirect:** Single‐cell RNAseq/spatial association	Skeletal Muscle	Fibroadipogenic progenitors (chemically induced senescence)	(**X. Zhang et al.** 2022)
*CX3CR1, CXCL14, CCL12, CCL2, CCL7, CXCL16, CCL5, CCR1, CCR10*	↑	**Indirect:** Single‐cell RNAseq/spatial association	Skeletal Muscle	Fibroadipogenic progenitors (senescent tissue‐resident, chronologically aged)	(**X. Zhang et al.** 2022)
*Cxcl3, Cxcl14*	↑	**Indirect:** Single‐cell RNAseq	Skeletal Muscle	Fibroadipogenic progenitors (senescent tissue‐resident, chronologically aged)	(Hettinger et al. 2022)
Ccl2, Ccl7, Ccl8‐Ccr2	↑	**Direct:** Therapeutically actionable (Ccr2) and transplantation	Skeletal Muscle	Fibroadipogenic progenitors (senescent tissue‐resident, chronologically aged)	(Blanc et al. 2020)
**Cardiac**					
*Ccl2*, *Ccl8*, *Cxcl12*, and *Cx3cl1*	↑	**Indirect:** Transcriptome SASP profiling	Cardiac	Cardiac MSCs (senescent tissue‐resident, chronologically aged)	(Martini et al. 2019)
**Hair follicle**					
*Ccl2, Cxcr4*	↓	**Direct:** Therapeutically actionable, functional‐coculture and transplantation	Hair follicle	Dermal Papilla (senescent, tissue‐resident, chronologically aged)	(W. Wu et al. 2023)
*Cxcl9, Cxcl10, Ccl3, Ccl7, Ccl8*	↓	**Indirect:** Single‐cell RNAseq	Hair follicle	Dermal Papilla (senescent tissue‐resident, chronologically aged)	(W. Wu et al. 2023)
*CCL2, CXCL8*	↑	**Indirect:** Single‐cell RNAseq, protein levels (CCL2, CXCL8)	Hair follicle	Dermal Papilla (in vitro expanded, replicatively senescent)	(**Pappalardo et al.** 2025)
**Dental pulp**					
CCL11	↑	**Direct:** Therapeutically actionable, functional‐coculture and transplantation	Dental pulp	Dental pulp MSC (senescent tissue‐resident, chronologically aged and inflammatory stress induced)	(**Zayed et al.** 2020)
**Lung**					
Ccl12/Mcp‐5, CCL11/Eotaxin, Ccl2/Mcp‐1, Cxcl2/Mip‐2	↑	**Direct:** Functional co‐culture	Lung	Lung resident MSCs (senescent tissue‐resident, chronologically aged)	(Chanda et al. 2021)

*Note:* Chemokines reported in primary studies are organized by stem cell niche and MSC type. Changes in expression are indicated as increased (↑) or decreased (↓). Evidence is classified as indirect (secretome or transcriptomic profiling) or direct (genetic or pharmacologic targeting), and the primary experimental system is summarized. Gene symbols follow species‐specific capitalization and are italicized; protein names are not. Human studies are shown in bold.

### 2.3 Homeostatic Chemokine Programs Maintain Stem Cell Function

Homeostatic chemokine programs comprise spatially and temporally regulated signals synthesized by functional MSCs to coordinate stem cell retention, quiescence, activation, migration, and self‐renewal. Their biological effects depend on ligand abundance, spatial localization, duration of production, and the receptor repertoire of responding cells. Aging can compromise these programs through depletion of functional MSC populations, reduced synthesis of supportive chemokines, or impaired chemokine sensing.

MSCs are fundamental components of the BM niche and play essential roles in the maintenance and regulation of HSCs through both physical interactions and paracrine signaling (Crippa and Bernardo 2018; Kfoury and Scadden 2015; Matsuoka et al. 2015; Méndez‐Ferrer et al. 2010; Sugiyama et al. 2006; Wagner et al. 2007). The BM niche is the most extensively characterized MSC‐supported stem cell system and is used throughout this review as a reference point for mechanisms that are comparatively less established elsewhere. Among BM MSC subpopulations, CXCL12‐abundant reticular (CAR) cells, many of which express the intermediate filament protein Nestin, are a critical niche component. CAR cells express high levels of CXCL12, also known as stromal‐derived factor 1 (SDF‐1), which is essential for hematopoietic stem cell (HSC) retention and quiescence within the BM niche (Aoki et al. 2021; Sugiyama et al. 2006). CAR cell‐derived CXCL12 maintains neighboring HSCs in a quiescent state through CXCR4 expressed on HSCs (Miao et al. 2022; Sugiyama et al. 2006). Multipotent BM MSCs also give rise to osteoblasts, which contribute to HSC development, differentiation, and long‐term maintenance (Crippa and Bernardo 2018; Kfoury and Scadden 2015; Singh et al. 2020).

Homeostatic CXCL12–CXCR4 signaling also regulates MSC function. CXCR4 is required for MSC homing and migration, and its downregulation in aged MSCs is associated with reduced chemotaxis and motility, limiting their ability to respond to tissue damage and niche perturbation (Kyriakou et al. 2008; Sanghani‐Kerai et al. 2017; Shao et al. 2011). Loss of CXCR4 signaling also impairs the osteogenic potential of aged BM MSCs, linking chemokine sensing to both niche support and lineage specification (Singh et al. 2020). The suture mesenchyme represents a distinct craniofacial niche for resident skeletal stem cell MSCs. Within this niche, constitutive and injury‐induced CXCL12 acts on CXCR4‐expressing Gli1+ suture stem cells to promote their expansion and directional migration and to induce Shh/Ihh‐dependent osteoblastic differentiation, whereas disruption of this signaling impairs calvarial regeneration (B. Li et al. 2023, 2025). The suture mesenchyme also undergoes age‐associated inflammatory and mesenchymal remodeling, consistent with broader evidence that intrinsic skeletal stem cell aging generates a pro‐inflammatory, poorly regenerative skeletal niche (Ambrosi et al. 2021; B. Li et al. 2023).

Single‐cell transcriptional profiling has provided additional insight into age‐associated alterations in the BM MSC compartment. Khong and colleagues identified a distinct quiescent MSC population in young human BM with reduced expression of proliferation‐ and differentiation‐associated genes; this stem cell‐like population was absent from aged samples, suggesting an age‐related loss of MSC self‐renewal (Khong et al. 2019). Functionally, aged MSCs exhibited reduced ability to promote repair in a murine excisional wound model compared with young MSCs, indicating that depletion of this quiescent population compromises MSC‐mediated regenerative potential.

Other tissues contain specialized MSC populations that similarly provide homeostatic paracrine, chemotactic, and structural support. In skeletal muscle, fibroadipogenic progenitors (FAPs) represent a specialized MSC population that regulates muscle stem cell activation, expansion, and differentiation (Theret et al. 2021; X. Zhang et al. 2024). FAPs reside within the muscle stem cell niche and are maintained in a quiescent state under homeostatic conditions, rapidly proliferating after injury to support tissue repair (Joe et al. 2010). They are strategically positioned within connective tissue compartments, including perineural regions and neuromuscular junctions, and engage in reciprocal interactions with macrophages to coordinate inflammation resolution and tissue regeneration (Theret et al. 2021). Although FAPs do not directly contribute to myogenic lineages, they are essential for maintaining satellite cell function and regulating the tempo of myogenic differentiation (Contreras et al. 2021; Heredia et al. 2013; Kurosawa et al. 2024; Mozzetta et al. 2013; Uezumi et al. 2014, 2021).

MSCs have also been isolated from dental pulp, deciduous teeth, periodontal ligament, apical papilla, and dental follicles. Within dental pulp, MSCs coexist with dental pulp stem cells and neural crest‐derived progenitors, collectively supporting pulp regeneration through trophic and chemotactic factors such as CCL11, CCL25, CXCL14, and MCP‐1/CCL2 (Kurogoushi et al. 2021; Matsumura‐Kawashima et al. 2021; Noda et al. 2019; Zayed et al. 2020). Dental pulp MSCs can enhance stem cell proliferation, reduce senescence markers, and improve osteogenic differentiation capacity in co‐culture (Ma et al. 2019). These MSCs may also be more resistant to replicative and inflammation‐induced senescence than BM‐derived MSCs, highlighting tissue‐specific differences in MSC aging trajectories (Ma et al. 2019).

The hair follicle stem cell niche similarly depends on reciprocal interactions between epithelial and mesenchymal populations to support cyclical regeneration through growth, regression, and quiescence (Fernandes et al. 2004; Ji et al. 2017; B. Wang et al. 2020). The dermal papilla contains multipotent MSC‐like populations that function as a central signaling compartment for hair follicle morphogenesis and cycling (Bai et al. 2017; Ji et al. 2017; Rahmani et al. 2014; B. Wang et al. 2020). Additional NG2‐ and CD34‐expressing MSC‐like cells surround the hair follicle, suggesting a broader mesenchymal support network (Yamanishi et al. 2012). Following follicle injury, *Ccl2, Ccl3, Ccl4, Ccl9, Cxcl2, Cxcl11, and Cxcl13* contribute to hair follicle stem cell reactivation and self‐organization in murine models (W. Wu et al. 2023).

Lung‐resident MSCs localize to the vascular adventitia, perivascular alveolar interstitium, and stromal regions of pulmonary arteries, positioning them at interfaces between the circulation and tissue parenchyma (Doherty et al. 2023; Steens et al. 2021; Sveiven and Nordgren 2020). Mesenchymal populations provide paracrine and structural support to Type II alveolar epithelial cells, which serve as the principal stem cell population responsible for alveolar renewal (Allen et al. 2022; Liao and Li 2020). Lung‐resident MSCs expressing SCGB1A1 and integrin α6β4 have been identified within airway and alveolar regions, further supporting their niche‐defining roles (Chapman et al. 2011; Rawlins et al. 2009).

Cardiac MSCs constitute an abundant mesenchymal population within the healthy human heart and contribute to stromal maintenance and vascular support (Pilato et al. 2018). Following myocardial injury, these cells are activated and recruited to sites of damage through mechanisms that include CXCL12‐CXCR4 signaling (Zhuang et al. 2009). Cardiac MSCs can also give rise to endothelial and smooth muscle lineages that support tissue homeostasis and repair (Martini et al. 2019).

Within the neural niche, the neurovascular unit integrates endothelial cells, neurons, astrocytes, and perivascular MSC‐like cells to regulate stem cell behavior (Appaix et al. 2014; Kaminska et al. 2022). Perivascular cells in the brain, often classified as pericytes, have been reported to share some phenotypic and functional characteristics with MSCs, although heterogeneity in isolation methods and marker expression means this classification remains unresolved (Crisan et al. 2008; Lojewski et al. 2015; Özen et al. 2012). Perivascular MSCs are well positioned to regulate neural stem cells through paracrine signaling, angiogenic support, and immunomodulation (Kaminska et al. 2022; Salgado et al. 2015). In vitro, MSCs secrete neuroprotective and pro‐angiogenic factors that influence neural stem cell proliferation, differentiation, and survival (Hsieh et al. 2013; Oppliger et al. 2017; Salgado et al. 2015; Y. Wang et al. 2009). CXCL12–CXCR4 signaling contributes to neural stem cell proliferation and maintenance, illustrating conservation of this homeostatic pathway beyond the BM (Chaudhary et al. 2025; Zhu et al. 2019).

Together, these examples demonstrate that MSC‐derived chemokines are normal components of tissue maintenance and repair. Chemokines associated with inflammatory recruitment can perform homeostatic functions when their production is appropriately localized and resolves after injury. The distinction between homeostatic and pathological signaling therefore lies not solely in chemokine identity, but in the abundance, location, duration, and cellular context of its synthesis.

### 2.4 Senescence‐Associated Chemokine Synthesis Disrupts Signaling Fidelity

With aging, MSC chemokine synthesis shifts from tightly regulated homeostatic programs toward profiles associated with chronic senescence and the SASP. We use “senescence‐associated” where altered chemokine synthesis has been observed in aged or senescent MSCs, but a direct causal relationship has not been established. This transition includes both loss of supportive chemokine signals and gain of persistent inflammatory signals.

The CXCL12–CXCR4 axis provides direct evidence that disruption of homeostatic chemokine signaling contributes to stem cell aging. BM MSCs from aged mice exhibit reduced *Cxcr4* expression, resulting in impaired regulation of HSC fate (Singh et al. 2020). Co‐culture of young HSCs with *Cxcr4*‐deficient aged BM MSCs induced premature HSC aging, characterized by reduced self‐renewal and impaired differentiation capacity. Targeted deletion of *Cxcr4* in young mice was sufficient to recapitulate an aged HSC phenotype, providing genetic evidence that *Cxcr4* expression within the BM environment is required for HSC maintenance. BM MSCs from aged mice also exhibit reduced expression of *Cxcl12*/SDF‐1 (Guang et al. 2013).

Neural niches exhibit both loss of supportive chemokine signaling and attrition of mesenchymal support populations. CXCL12–CXCR4 signaling diminishes with age, contributing to reduced neurogenesis and impaired neural stem cell regulation (Chaudhary et al. 2025; Zhu et al. 2019). MSCs are a source of CXCL12 both in vitro and in vivo, suggesting that age‐associated MSC dysfunction could contribute to this chemokine insufficiency (Kitaori et al. 2009; X. Wang et al. 2021). Aging brain pericytes undergo progressive loss associated with vascular instability, blood–brain barrier disruption, neuroinflammation, and cognitive decline (Bell et al. 2010). Brain‐wide transcriptional analyses further identify pericytes as one of the most significantly depleted populations across aging brain regions (Jin et al. 2025).

BM MSCs from aged donors simultaneously acquire a remodeled secretory profile that impairs HSC function. Exposure of young HSCs to soluble factors produced by aged BM MSCs reduces their clonogenic capacity and induces pro‐inflammatory gene expression (Gnani et al. 2019). Aged BM MSCs exhibit reduced expression of the immunomodulatory chemokine *CXCL10* alongside increased *CCL2/*MCP‐1 and the inflammatory cytokines *IL‐1β* and *IL‐6*. Their conditioned media also contain increased IL‐8/CXCL8, CCL4, CXCL2/GRO‐β, and CCL/2MCP‐1, together with the SASP cytokines IL‐1α and IL‐6. Secretion of IL‐6 and CXCL8 by replicatively aged BM MSCs disrupts MSC‐mediated maintenance of HSC quiescence, thereby accelerating exhaustion of the HSC compartment (O'Hagan‐Wong et al. 2016).

A comparable gain of inflammatory chemokine synthesis occurs in the aging musculoskeletal system. Within osteoarthritic joints, senescent synovial MSCs acquire a SASP characterized by increased IL‐8/CXCL8, CXCL1, CXCL5, and CXCL6, together with IL‐1β and IL‐6 (Cao et al. 2019; Lehmann et al. 2022; Siraj et al. 2023). Increased production of these factors promotes synovial inflammation, cartilage fibrosis, and progressive joint degeneration (Copp et al. 2023; W. Liu et al. 2021; Sekelova et al. 2023; S. Zhou et al. 2008; Zupan 2021). Senescence and SASP expression within osteoarthritic joints are largely confined to MSC progenitor populations rather than their chondrocyte progeny, suggesting that senescent MSCs act upstream of cartilage degeneration by altering the environment that supports cartilage maintenance (W. Liu et al. 2021).

In skeletal muscle, aged FAPs exhibit diminished capacity to support muscle stem cell expansion and differentiation (L. Liu et al. 2022; Lukjanenko et al. 2019). Transcriptomic analyses in mice reveal age‐associated activation of chemokine pathways in FAPs, including increased *Ccl2* and *Ccl7* expression (X. Zhang et al. 2022). Aged human skeletal muscle similarly exhibits increased *CX3CR1, CXCL14, CCL12, CCL2, CCL7, CXCL16, CCL5, CCR1*, and *CCR10* expression (X. Zhang et al. 2022). During disuse atrophy, aged murine FAPs are enriched for chemotaxis‐associated genes, including *Cxcl3* (*CXCL1* in humans) and *Cxcl14*, in a tissue environment characterized by immune‐cell infiltration, increased ECM deposition, fibrosis, and accelerated depletion of the muscle stem cell pool (Hettinger et al. 2022). Signaling through Ccr ligands such as Ccl2, Ccl7, and Ccl8 promotes progenitor differentiation but becomes dysregulated with age, as evidenced by increased Ccr2 expression in aged mouse muscle (Blanc et al. 2020).

Senescence‐associated chemokine synthesis also affects the organization of epithelial stem cell compartments. Hair follicle aging is characterized by depletion and dysfunction of dermal papilla cells and accumulation of senescent cells within the mesenchymal compartment (J. H. Lee and Choi 2024; Shin et al. 2020). Aged hair follicle stem cells exhibit impaired regeneration, prolonged quiescence, aberrant differentiation, or loss of niche identity (W. Wu et al. 2023). These changes occur within an inflammatory environment containing TNF‐α, IL‐1β, CXCL9, and CXCL13 (Doles et al. 2012; Hu et al. 2017). Aged hair follicle stem cells also display reduced niche mobilization and regenerative potential in transplantation assays, which may reflect altered chemokine gradients or responsiveness as contributing factors (Ge et al. 2020). Transcriptomic analysis of aged murine hair follicles identified senescence‐ and SASP‐associated genes within mesenchymal lineage cells, with dermal papilla cells exhibiting particularly strong SASP signatures and SA‐β‐gal (Shin et al. 2020). Serially passaged human dermal papilla cells similarly accumulate IL‐8/CXCL8 and MCP‐1/CCL2, together with IL‐6 (Pappalardo et al. 2025).

Aging of the pulmonary niche is accompanied by accumulation of inflammatory and senescent cells, producing a signaling environment that overlaps with the SASP (Allen et al. 2022). MSCs isolated from lungs of older patients with severe COVID‐19 exhibited increased expression of chemokines associated with inflammation and immune‐cell recruitment, including *CCL5*, *CCL2*, and *CCL3* together with other transcriptional features consistent with senescence (Henriques‐Pons et al. 2022). In an alveolosphere model, lung‐resident MSCs from young mice supported epithelial organoid self‐assembly, whereas aged MSCs secreted increased Ccl12/Mcp‐5, Ccl11/Eotaxin, Ccl2/Mcp‐1, and Mip‐2 and disrupted alveolar stem cell organization (Chanda et al. 2021). This system provides functional evidence that the secretory state of aged MSCs can directly compromise epithelial regeneration.

In other murine models of cardiac aging, a distinct population of pro‐inflammatory MSCs exhibits increased expression of senescence‐associated genes, including *Cdkn2a* and *Cdkn2b* (Martini et al. 2019). These natively aged murine MSCs express increased *Ccl2*, *Ccl8*, *Cxcl12*, and *Cx3cl1*, contributing to a gene expression landscape of a chronically inflamed myocardial environment. Although young and aged cardiac MSCs retain endothelial differentiation capacity, aged MSCs exhibit reduced expression of endothelial markers, linking their altered secretory state to a broader loss of vascular regenerative potential (Martini et al. 2019).

Collectively, these studies do not identify a single conserved senescence‐associated chemokine repertoire. Instead, they reveal a conserved loss of signaling fidelity. Reduced synthesis of supportive chemokines and impaired receptor‐mediated sensing coexist with persistent production of inflammatory and chemotactic ligands. These changes alter direct MSC–stem cell communication, recruit immune populations, and disrupt the cellular and spatial organization required for tissue regeneration. The strength of evidence varies across tissues. Functional co‐culture or transplantation studies link altered MSC chemokine signaling to stem cell outcomes in the hair follicle, dental pulp, lung, and the bone marrow CXCL12–CXCR4 axis. Evidence in skeletal muscle and the cranial suture is based mainly on single‐cell and spatial analyses, whereas evidence in the cardiac niche remains indirect (Table 2). These differences likely reflect how extensively and readily each niche has been studied rather than its biological importance.

### 2.5 Chemokine Pathways as Candidate Therapeutic Targets

Therapeutic actionability represents a third, overlapping dimension of the model. A homeostatic or senescence‐associated pathway becomes therapeutically actionable when manipulation of the ligand, receptor, or cellular source modifies a relevant regenerative outcome. The appropriate intervention depends on the underlying defect and may require restoration of a depleted homeostatic signal, inhibition of a persistent senescence‐associated signal, or normalization of a pathway that has shifted from an adaptive to a maladaptive function.

The CCL11–CCR3 axis provides direct evidence that inhibition of an age‐associated chemokine pathway can restore regeneration. CCL11/Eotaxin is elevated in chronic inflammatory conditions and has been proposed as a circulating marker of aging (Hoefer et al. 2017; Kurogoushi et al. 2021; Zayed et al. 2020). In a canine model of tooth regeneration, CCR3 antagonism restored pulp regeneration in aged tissue, as evidenced by enhanced dentin formation, neurite outgrowth, and angiogenesis (Zayed et al. 2020). Complementary analysis of human periodontal ligament stem cells demonstrated that senescence‐associated *Ccl11* upregulation coincided with increased *IL‐8, IL‐1β*, and *p21* expression when senescence was induced by p‐Cresol, whereas CCR3 antagonism attenuated inflammatory signaling and enhanced immunomodulatory marker expression. These findings support a functional relationship between CCL11–CCR3 signaling and regenerative decline, although the contribution of MSC‐derived CCL11 to the in vivo response remains to be established.

The CXCL12–CXCR4 axis represents a second candidate target, but its context‐dependent functions require different therapeutic approaches between tissues. Reduced CXCL12–CXCR4 signaling compromises HSC maintenance, MSC migration, osteogenic differentiation, and neural stem cell support, suggesting that preservation or restoration of this pathway may be beneficial in BM and neural tissues (Chaudhary et al. 2025; Guang et al. 2013; Singh et al. 2020; C. Zhu et al. 2019). By contrast, CXCL12 has been reported to inhibit hair growth through CXCR4–STAT signaling, suggesting that excessive or persistent activity may suppress follicular regeneration (Zheng et al. 2022). The evidence does not establish that this effect arises specifically from aged MSCs, but it illustrates why therapeutic manipulation should aim to restore tissue‐appropriate CXCL12–CXCR4 signaling rather than uniformly activate or inhibit the pathway.

An example of direct restoration of a depleted homeostatic chemokine is illustrated by CCL2 signaling in skin. RNAseq analysis showed that chemokine‐pathway genes, including CCL and CXCL family members, were decreased in adult relative to newborn mouse skin organoid cultures, with particularly lower *Ccl2* and *Ccr4* expression (W. Wu et al. 2023). Addition of recombinant CCL2 to adult skin cell organoids increased epidermal aggregate formation, maintained cell multipotency, enhanced epithelial–mesenchymal organization, and increased hair follicle regeneration after transplantation. However, CCL2 was tested as part of an environmental reprogramming protocol in which both control and treatment cultures also received Wnt10a, MMP14, and a PKC inhibitor; thus, these findings identify CCL2 as a contributing regenerative signal rather than establish that CCL2 alone is sufficient. Moreover, the cultures contained epithelial, dermal, and immune populations, and the relevant source and responding cells were not restricted to MSCs. Nevertheless, the study provides proof of principle that direct restoration of a chemokine signal diminished in adult tissue can improve regenerative competence without broadly eliminating senescent cells.

Senolytic and senomorphic strategies provide a broader, source‐directed means of modifying multiple senescence‐associated chemokines simultaneously. Treatment of aged mice with dasatinib and quercetin improved the proliferation and osteogenic capacity of BM MSCs while reducing SASP‐associated *Il‐6*, *Cxcl1* (*CXCL3* in humans), and *Ccl2* expression (Y. Zhou et al. 2021). In a progeroid mouse model, fisetin selectively eliminated senescent FAPs, reduced *Il‐1α, Il‐1β, Cxcl1* (*CXCL3* in humans), and *Ccl1* expression, and restored muscle progenitor proliferation and differentiation (L. Liu et al. 2022). These studies support the principle that targeting the senescent cellular source can normalize the chemokine environment. However, because senotherapeutics modify numerous SASP components simultaneously, they do not establish that suppression of an individual chemokine accounts for the functional improvement.

Translation is further complicated by the fact that senescent MSCs do not comprise a single targetable state. Their markers, SASP composition, survival dependencies, and senolytic sensitivity vary with tissue source, donor characteristics, senescence‐inducing stress, and duration of senescence (Basisty et al. 2020; Kirkland and Tchkonia 2020; Suryadevara et al. 2024). Current agents such as dasatinib plus quercetin, fisetin, and navitoclax were not developed to recognize MSC lineage, and no clinically validated MSC‐specific senolytic or universal marker of senescent MSCs is available. In cultured human adipose‐derived MSCs, CD26‐high cells exhibited reduced immunopotency and were reduced by navitoclax, high‐dose quercetin, and 17‐DMAG. However, all of these treatments also reduced overall MSC viability, and the analysis was limited to replicative senescence in a small donor cohort (Psaroudis et al. 2022). Thus, apparent senolysis in culture does not establish selective removal of pathogenic senescent MSCs in vivo.

Broad clearance may also remove transient senescent stromal populations that participate in acute tissue repair or constrain fibrosis. For example, CCN1‐induced fibroblast senescence activated antifibrotic programs and limited fibrosis during cutaneous wound healing (Jun and Lau 2010) although whether comparable reparative senescence occurs within MSC subsets is not yet known. The timing and route of senotherapeutic delivery are therefore central to the therapeutic index. Systemic senolysis may be attractive when senescent‐cell burden and SASP signaling are distributed across organs, but it increases exposure of non‐target cell populations and cannot distinguish senescent MSCs from other senescent cells. Local delivery could concentrate clearance or SASP modulation within a diseased niche and preserve reparative senescence elsewhere, but it may not correct circulating SASP factors or multisystem inflammaging and may be impractical for inaccessible tissues. Senomorphics may preserve viable MSCs while reducing harmful secretory output, but they may require repeated or sustained administration and could also interfere with adaptive, injury‐responsive signaling. There are few direct comparisons of local and systemic senotherapeutic delivery in MSC‐aging models, which warrants further study.

These limitations provide a rationale for targeting individual SASP mediators or chemokine–receptor pathways when a causal signal can be defined. Neutralization of IL‐1α and IL‐8/CXCL8 rescued the clonogenic and osteogenic capacity of early‐passage MSCs exposed to late‐passage MSCs (Chou et al. 2022), whereas neutralization of IL‐6, IL‐8/CXCL8, or CCL2 enhanced the T‐cell‐suppressive capacity of adipose‐derived MSCs from older patients with atherosclerosis (Kizilay Mancini et al. 2017). The latter result contrasts with the regenerative effect of CCL2 supplementation in adult skin organoids (W. Wu et al. 2023), illustrating that the same chemokine may function as a depleted regenerative signal in one setting and an excessive, dysfunction‐associated signal in another. Chemokine‐directed treatment may therefore be more mechanistically precise than broad senescent‐cell clearance because it can preserve the MSC population and its non‐chemokine reparative functions. It should not, however, be assumed to be inherently safer. Chemokines are pleiotropic and redundant, and systemic agonism or antagonism may disrupt immune recruitment, host defense, or homeostatic trafficking beyond the intended niche. Accordingly, the ligand or receptor selected, the direction of modulation, the treatment window, and local versus systemic delivery must be matched to the tissue and disease state.

The three programs are therefore best understood as intersecting dimensions rather than discrete molecular classes. Homeostatic and senescence‐associated programs describe the functional state of MSC‐derived signaling, whereas therapeutic actionability describes whether that state can be modified (Table 1). Therapeutic benefit is therefore most likely to arise from restoring the appropriate identity, abundance, location, and duration of chemokine signaling rather than broadly suppressing chemokine activity: direct chemokine restoration or blockade when a causal signaling defect has been established; senomorphic intervention when several interacting SASP components drive dysfunction but preservation of the MSC compartment is desirable; and senolysis when a persistent, pathogenic senescent‐cell population has been demonstrated and can be cleared without unacceptable loss of reparative cells.

### 2.6 Restoring MSC Function as an Upstream Strategy for Niche Rejuvenation

MSC‐based therapies have emerged as a potential intervention for degenerative diseases associated with chronological aging (Kabat et al. 2020; Lalu et al. 2012; Lu and Allickson 2025; Martin et al. 2017; Renesme et al. 2022; Sharma et al. 2014; Wiese et al. 2022). However, patient‐derived MSCs may be a suboptimal therapeutic source because age‐associated functional decline can limit the large‐scale expansion required to achieve therapeutic doses (Bijonowski et al. 2020; Miclau et al. 2023; X. Wang et al. 2023; Wiese et al. 2019). Strategies that restore aged MSCs toward a more youthful state or replace dysfunctional tissue‐resident MSC populations have therefore emerged as complementary approaches to niche rejuvenation.

Tissue‐resident MSCs are highly responsive to local matrix stiffness, topology, and biochemical composition and actively remodel the ECM, capacities that diminish with age (Burk et al. 2022; Mauney et al. 2010; Park et al. 2023). Remarkably, both murine and human aged BM MSCs cultured on ECM deposited by young MSCs shifted toward a more youthful phenotype, suggesting that structural and soluble cues retained within young ECM can support stemness and suppress senescent features (Block et al. 2017; Y. Sun et al. 2011). Hung and colleagues demonstrated that conditioned media from young murine BM MSCs contained paracrine signals that restored the angiogenic capacity of aged MSCs (Hung et al. 2022). Although these studies did not determine whether altered chemokine synthesis mediated the observed effects, they demonstrate that age‐associated MSC dysfunction is not necessarily irreversible and can be modified by extracellular and paracrine cues.

Direct cell–cell interactions further contribute to niche‐mediated restoration of stem cell function. For example, transplantation of young FAPs into aged muscle restored myogenic stem cell function through secretion of WISP‐1, a matricellular protein required for muscle regeneration and stem cell fate regulation (Lukjanenko et al. 2019). Similarly, addition of young lung‐resident MSCs restored the self‐assembly and function of aged alveolar epithelial stem cell organoids (Chanda et al. 2021). In the same experimental system, aged lung MSCs produced increased CCL12, CCL11, CCL2, and CXCL2 protein and disrupted alveolar stem cell organization. Restoration by young MSCs is therefore consistent with correction of an age‐associated paracrine environment, although it does not establish that normalization of these chemokines alone accounts for the regenerative effect.

Together, these studies support the capacity of youthful or functionally restored MSCs to rejuvenate aging stem cell microenvironments through coordinated regulation of ECM composition, paracrine signaling, and direct cellular interactions. These effects extend beyond chemokines and should not be interpreted as evidence that chemokine normalization is the sole mechanism of MSC‐mediated rejuvenation. Rather, they position the functional state of the MSC as an upstream regulator of multiple niche‐supportive programs, including chemokine synthesis. Rejuvenating the MSC compartment may therefore complement direct targeting of individual chemokine–receptor axes by simultaneously restoring homeostatic signals and reducing senescence‐associated signaling.

Despite this promise, MSC‐based rejuvenation carries additional translational limitations. Improvement in selected markers or functions ex vivo does not establish durable rejuvenation after cells are returned to an aged, inflammatory, or mechanically altered niche, and expansion to therapeutic doses can itself promote replicative senescence and functional drift (Psaroudis et al. 2022; Wiese et al. 2019). Delivery route further shapes the expected mechanism. Preclinical studies show that intravenously infused MSCs are largely retained in the pulmonary microvasculature and are short‐lived, limiting direct access to many distant niches and suggesting that systemic effects may instead be mediated indirectly (Braid et al. 2018; Eggenhofer et al. 2014; Shokoohmand et al. 2026). Local administration may increase exposure within a defined tissue but is less suited to multisystem aging and may require repeated delivery. Clinical measurement is equally challenging: changes in circulating SASP factors cannot be attributed specifically to tissue‐resident MSCs, whereas serial biopsies and direct functional assays are invasive and tissue‐dependent. Because no single marker identifies senescence across tissues, evidence of MSC rejuvenation should combine operational identification of the relevant tissue‐resident MSC population with multiple senescence hallmarks, restoration of tissue‐appropriate chemokine signaling and MSC function, and a regenerative or clinical endpoint (Ogrodnik et al. 2024; Suryadevara et al. 2024). Until such measures are validated, “MSC rejuvenation” should be interpreted as restoration of selected MSC functions rather than reversal of biological age.

## 3 Conclusion

MSCs regulate stem cell niches through architectural, signaling, and inflammatory functions. Aging depletes supportive MSCs and accumulates senescent MSCs with altered secretory profiles. Within the synthesis‐based model proposed here, MSC senescence occupies an intermediate, amplifying position: it arises downstream of molecular, metabolic, epigenetic, and inflammatory stress and translates these inputs into altered chemokine outputs that reshape the signaling environments governing stem cell function. These changes amplify chronic inflammation, disrupt intercellular communication, and contribute to stem cell exhaustion and regenerative decline. The evidence comprises two intersecting signaling states—homeostatic and senescence‐associated—with therapeutic actionability as a third dimension. Aging depletes chemokines needed for stem cell retention, quiescence, migration, and repair while increasing signals that recruit immune cells and reinforce chronic inflammation.

Across tissues, no single chemokine repertoire defines MSC aging. Instead, a common loss of signaling fidelity alters chemokine identity, abundance, location, duration, and receptor‐mediated sensing. This disrupts direct MSC–stem cell communication and broader immune, vascular, and stromal networks, contributing to stem cell exhaustion, impaired differentiation, disorganized tissue, and declining regeneration. Environmental and metabolic stress further reinforce this transition by accelerating MSC senescence and embedding systemic aging signals in local niches.

The plasticity of stem cell niches provides opportunities for intervention. Therapeutically actionable pathways may be addressed by restoring deficient homeostatic signals, inhibiting persistent senescence‐associated signals, or targeting the dysfunctional MSCs that produce them. Because chemokines can be beneficial or detrimental depending on tissue context, therapy should normalize chemokine signaling rather than uniformly activate or suppress a pathway. Senotherapeutic approaches may reduce multiple senescence‐associated signals, whereas rejuvenating or replacing aged MSCs may restore homeostatic chemokine synthesis alongside other supportive functions.

Importantly, many rejuvenation and senotherapeutic studies modify multiple components of the MSC secretome and do not establish that changes in an individual chemokine mediate the observed functional improvement. Future studies must therefore distinguish chemokines associated with MSC aging from those that are necessary or sufficient to drive stem cell dysfunction and determine whether their normalization restores regenerative function in vivo. Collectively, the evidence positions dysregulated chemokine synthesis by tissue‐resident MSCs as a mechanistic interface between cellular aging, chronic inflammation, and stem cell decline, while identifying restoration of MSC signaling fidelity as a potential strategy to preserve regenerative capacity during aging.

## Author Contributions

**Shaianne N. Stein:** collection and/or assembly of literature, analysis and interpretation, manuscript writing, critical review of the manuscript and final approval of manuscript. **Lorena R. Braid:** conception/design, assembly of literature, analysis and interpretation, manuscript writing, critical review of the manuscript and final approval of manuscript.

## Funding

L.R.B. holds an NSERC Discovery Grant and a Canada Research Chair (Tier 2) in MSC Biology. These funding sources provided stipend support to S.N.S. during the preparation of this review.

## Conflicts of Interest

L.R.B. is founder and CSO of Aurora BioSolutions Inc. S.N.S. declares no conflicts of interest. The authors declare that the research was conducted in the absence of any commercial or financial relationships that could be construed as a potential conflict of interest.

## Data Availability

Data sharing not applicable to this article as no datasets were generated or analysed during the current study.
